# Simultaneous, Non-Contact and Motion-Based Monitoring of Respiratory Rate in Sheep Under Experimental Condition Using Visible and Near-Infrared Videos

**DOI:** 10.3390/ani14233398

**Published:** 2024-11-25

**Authors:** Beatriz Leandro Bonafini, Lukas Breuer, Lisa Ernst, René Tolba, Lucas Ferrari de Oliveira, Mauren Abreu de Souza, Michael Czaplik, Carina Barbosa Pereira

**Affiliations:** 1 Post Graduate Program in Technology in Health, Polytechnique School, Pontifical Catholic University of Paraná, Curitiba 80215-901, Brazil; mauren.souza@pucpr.br; 2 Department of Anesthesiology, Faculty of Medicine, RWTH Aachen University, 52074 Aachen, Germany; lubreuer@ukaachen.de (L.B.); mczaplik@ukaachen.de (M.C.); cbarbosapere@ukaachen.de (C.B.P.); 3 Institute for Laboratory Animal Science & Experimental Surgery, Faculty of Medicine, RWTH Aachen University, 52074 Aachen, Germany; lernst@ukaachen.de (L.E.); rtolba@ukaachen.de (R.T.); 4 Department of Informatics, Federal University of Paraná, Curitiba 81530-000, Brazil; lferrari@inf.ufpr.br

**Keywords:** animal welfare, respiration rate, sheep model, video signal processing, welfare assessment, refinement

## Abstract

The validation of methods for understanding the effects of many diseases and treatments requires the use of animal models in translational research. In this context, sheep have been employed extensively in scientific studies. However, the imposition of experimental conditions upon these animals may result in the experience of discomfort, pain, and stress. The ethical debates surrounding the use of animals in research have resulted in the adoption of Directive 2010/63/EU. The present study proposes a non-contact method for monitoring the respiration rate of sheep based on video processing. The Detecron2 model was trained to segment the sheep’s body, abdominal, and facial regions in the video frames. A motion-tracking algorithm was developed to assess abdominal movement associated with the sheep’s respiratory cycle. The method was applied to videos of Rhön sheep under experimental and housing conditions, utilising two types of cameras to assess the effectiveness of the proposed approach. The mean average error (MAE) obtained was 0.79 breaths/minute for the visible and 1.83 breaths/minute for the near-infrared (NIR) method. This study demonstrates the feasibility of video technology for simultaneous and non-invasive respiration monitoring, being a crucial parameter for assessing the health deterioration of multiple laboratory animals.

## Simple Summary

The concerns surrounding animal experimentation have been the subject of extensive discussions, resulting in the development of strategies that promote the adoption of non-invasive methods. This study aims to assess the viability of a non-contact method for estimating respiratory rate in sheep models in laboratory conditions. By segmenting the thoracic region and applying signal processing to detect frame-by-frame variations, we computed respiratory across various lighting conditions (visible and NIR cameras). The results demonstrated that the method is effective and provides a new methodology for assessing animal welfare, minimizing the experimental procedures impacts in pain, stress and discomfort in sheep.

## 1. Introduction

In translational scientific research, the quest for a comprehensive understanding of diseases and developing effective therapeutic strategies often requires animal models. Sheep models have taken on a significant role, underscored by features of research that emphasise their importance in the context of biomedical experimentation [1,2,3]. However, this fact raises significant ethical debates [4,5], where animals rely on human interest and diligence in understanding and respecting them while striving to improve their experimental conditions [6]. The concerns surrounding this issue prompted the adoption of the Directive 2010/63/EU by the European Union [7], aiming to improve animal welfare based on three fundamental principles (3Rs): replacement, reduction, and refinement [8]. The principle of replacement advocates using alternative or complementary methods in testing, thereby minimising the reliance on animal models. Reduction focuses on minimising the number of animals used in studies while ensuring the necessary scientific significance. Finally, refinement involves techniques and practices to reduce pain and suffering for these animals, such as employing less invasive methods or enhancing housing and care conditions. Therefore, pursuing methods that can measure coherent indicators with minimal human intervention is crucial. By doing so, it will be possible to balance scientific progress and animals’ ethical treatment in conducting similar research.

In contrast to the regulations aimed at minimising animal experimentation, the 2019 report issued by the European Union reveals that 20,000 sheep were employed for research purposes [9]. The use of sheep models in biomedical research is experiencing a growing trend, as this animal species has many similarities with human organ systems. According to a review by Banstola and Reynolds [10], research investigating human disorders using sheep models was divided by organs and systems, being: 2% focused on the eyes, 3% on the kidneys, 3% on endocrine systems, 7% on skin, 7% on musculoskeletal, 11% on immune systems, 15% on digestive systems, 15% on respiratory systems, 17% on the cardiovascular system, and 20% on the nervous system. The findings derived from sheep studies can indicate a noteworthy enhancement due to their physical stature, extended lifespan (enabling long-term treatment analysis), gestational period, and neuroanatomical resemblances to humans.

To comprehensively evaluate the health status of animals exposed to chronic experiments, it is essential to monitor their vital parameters objectively. Presently, telemetry systems are the standard for health monitoring [11]; however, such methods need initial surgical implantation of transmitters. This approach comes with several drawbacks. The implantation process can induce animal stress and pain, potentially leading to discomfort or injury. Furthermore, the implanted device may disrupt the animal’s natural behaviour, movement, and interaction with its environment. Additionally, it can exert significant adverse physiological effects, including inflammation, infection, tissue damage, alterations in blood flow, changes in hormonal and neurological responses, immune system reactions, and chronic pain. Lastly, transmitters have a short lifespan due to being powered by single-use batteries, which limits their use in animal studies [12].

In this context, efforts are underway to develop alternatives that allow contactless monitoring of vital parameters to predict early health deterioration. One method of assessing stress, pain, or discomfort involves observing vital parameters such as the respiration rate (RR). According to [13,14], in healthy sheep, RR ranges from 20 to 30 breaths per minute under normal conditions. Several factors can contribute to an increase or decrease in RR, including excitement caused by elevated environmental temperature, humidity, pain, fever, respiratory or cardiovascular disease, and respiratory compensation due to metabolic alterations [15]. It is important to emphasise that alterations in the animal’s environment and human interaction can also induce stress and result in variations in its physiological responses, being a recurring situation in experimental conditions.

These findings can be observed in Reefmann et al. [16], who investigated the physiological parameters, including RR, to alterations in sheep feeding patterns. In 2018, in turn, De et al. [17] evaluated the stress by isolating sheep while perceiving a significant change in RR. Cwynar and Kolacz [18] found a correlation between RR and acoustic stress in animals. Last but not least, the quantity of research concerning heat stress in sheep is remarkable, wherein most studies also employed RR as an evaluation parameter [19].

Some studies [20,21] showed that wearable sensors can measure RR in large animals. These devices are commonly used as collars and polar sensors [22]. However, it is crucial to acknowledge that the intervention necessary for applying these devices to the animal and the resulting discomfort may introduce alterations to the physiological data intended for subsequent analysis. The context of imaging and remote acquisition of vital signs has been widely addressed in research involving humans and animals. In addition, other authors [23,24,25] suggest methods for measuring RR through video recordings, where the frame-by-frame processing was able to indicate variations in pixel level caused by the respiratory and cardiac cycle. In the animal field, different species have been studied; for example, [26] performed RR extraction in pigs, while [27,28] in rodents, [29] in cattle, and [22] in sheep. Despite certain studies that have conducted the extraction of sheep’s RR, the analysis has been predominantly carried out based on thermal imaging processing, solely focusing on the temperature of the animal’s face.

Thus, this study’s principal contribution is the proposal of a non-contact method for extracting the RR in sheep, utilising both visible (RGB) and near-infrared (NIR) videos for the analysis of the chest/body.

## 2. Materials and Methods

**Figure 1:**
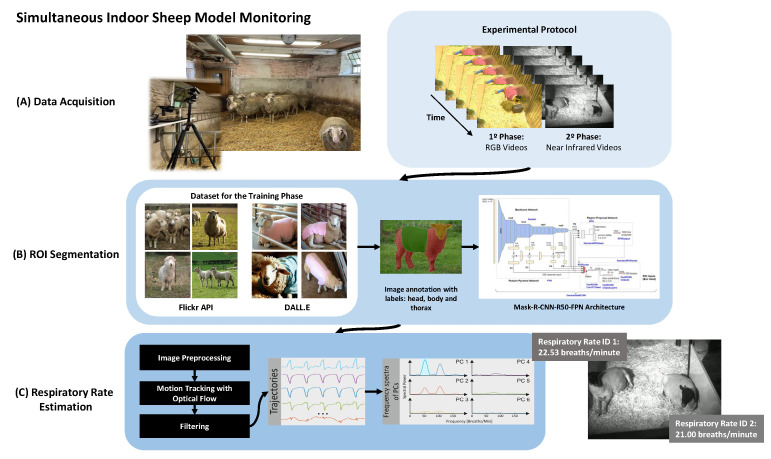
Schematic overview of the methods employed to extract RR in sheep using RGB and NIR videos.

This section presents an overview of the methodology employed in developing the motion-based algorithm capable of estimating RR in sheep. It considers the association between mechanical movements and the contraction and relaxation of muscles during the respiratory cycle. The method proposed is divided into three main stages: (A) data acquisition, (B) segmentation, and (C) RR estimation. Figure 1 shows an overview of the processing pipeline.

In the (A) data acquisition phase, videos of sheep were obtained for inclusion in the study, conducted in two different settings at the RWTH Uniklinik Animal Laboratory (Aachen, NRW, Germany). A total of six Rhön sheep were monitored throughout the study. The initial setting involved the acquisition of RGB videos of a single sheep under post-surgical and anaesthetised conditions. In the second setting, five additional sheep served as controls, with monitoring conducted using NIR imaging to capture data under varied lighting conditions. The use of these sheep aligns with the 3R principles, as they undergo invasive procedures, and the veterinarians aim to assess their health status and deterioration non-invasively. Section 2.1 provides in-detail video capture procedures.

The (B) region of interest (ROI) segmentation phase involved gathering sheep images from the Flickr [30] dataset and generating synthetic images via the DALL.E model [31]. These images constituted the training set for the automatic segmentation of the ROIs of the body parts in the videos captured of sheep within the laboratory environment, utilising the MaskRCNN model (schema provided by [32]). Further details are provided in Section 2.2. Finally, the (C) respiratory rate estimation phase involved calculating RR based on the segmented region from the previous phase, using thoracic movement detection through signal processing methods. These methods leveraged the frame-to-frame differences in visible (RGB) and near-infrared (NIR) video sequences (more detail in Section 2.3).

### 2.1. Data Acquisition—Experimental Protocol

The recordings were acquired in two different study stages. In the first stage, videos of laboratory sheep were recorded with an RGB camera, while in the subsequent stage, NIR videos were produced. Both studies were conducted at University Hospital Aachen (Aachen, Germany).

#### 2.1.1. First Stage—RGB Videos

The first stage adhered to the approved experimental protocol of the Governmental Animal Care and Use Committee of the federal state of North Rhine-Westphalia (LANUV, North Rhine-Westphalia, Germany)—No. AZ: 81-2.4.2020.251 and was conducted in compliance with the German Animal Welfare Law. All animals received humane care under the principles outlined in the “Guide for the Care and Use of Laboratory Animals” [33].

**Figure 2:**
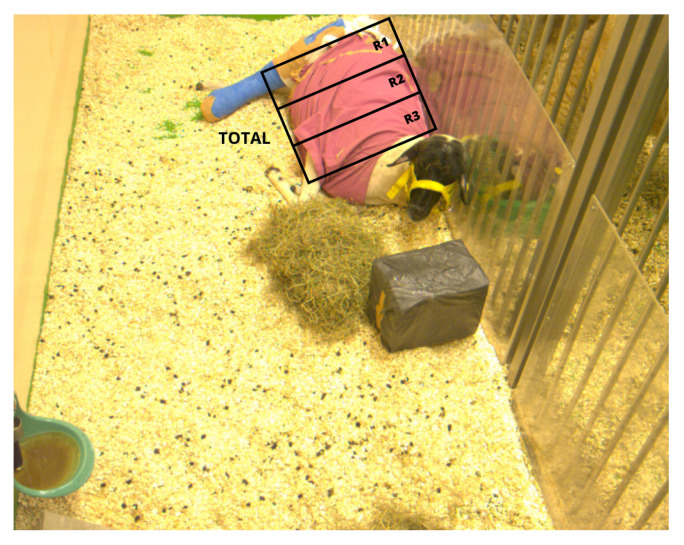
Sample of a frame collected during the experiments with the RGB camera. The figure shows a Rhön sheep resting with a cast on its leg and scrubs. A drawing also shows the regions of the sheep’s thorax corresponding to the R1, R2, R3, and total regions, which were later used to evaluate RR extraction.

In this initial stage, a single sheep was selected for inclusion, specifically a 4-year-old female Rhön sheep. Before the video capture, the sheep underwent a surgical procedure, and dressings were applied to its body, as shown in Figure 2. The sheep was housed in a room measuring approximately 3.1 m × 1.2 m. The RGB camera Basler acA1300-30gm (Basler AG, Ahrensburg, Germany) was used for video acquisition. The device presents a resolution of 1280 × 1024 pixels and a framerate of 20 fps. The camera was securely mounted on a tripod at an approximate distance of 2 m from the sheep. No additional illumination was provided. All videos were captured in raw format without any compression.

A pre-selection of the recorded videos was made to find only those moments when the sheep remained in a state of rest without interfering with any elements that might disturb the animal, such as handling or the presence of humans. Four videos were selected from this selection: three videos of 26 s and one video of 6 min and 3 s.

#### 2.1.2. Second Stage—NIR Videos

**Figure 3:**
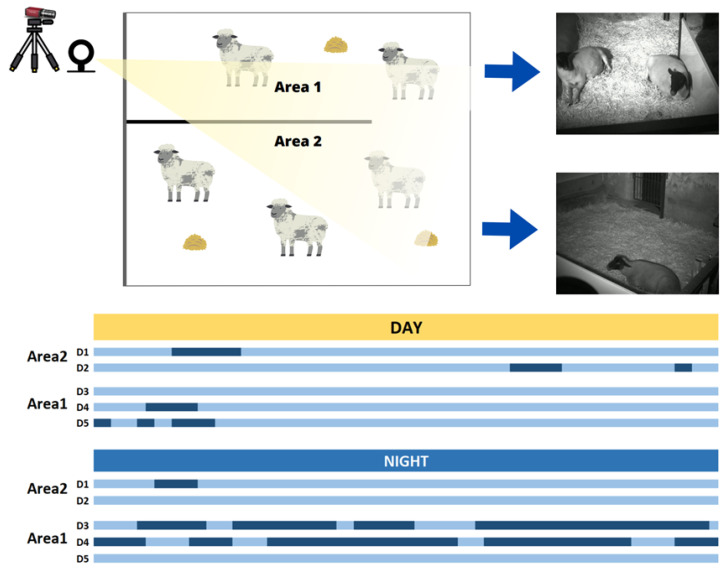
Schematic view of the second study stage showing the areas where the NIR videos were taken. The **top** of the figure shows which areas correspond to Area1 and Area2. Then, at the **bottom**, the time ranges at which sheep were at rest (dark blue). It is also presented the separation between day (7 a.m.) and night (7 p.m.).

For the second stage of the research, no approval for animal experiments was required as no experimental veterinary procedures were being performed on the animals; only videos were captured. Five female Rhön sheep (aged 6 to 8 years) were included, and the videos were collected over five nights from 22 to 26 July 2023. The sheep were already settled into their usual routine in a room with a total area of about 5.10 m × 3.7 m. In the middle of the room where the sheep were placed, a partition divided the region into two sections with the following dimensions: Area1—1.9 m × 3.7 m, Area2—3.2 m × 3.7 m. Figure 3 illustrates the measurement setup and displays for capturing frames from both areas for each respective zone. A NIR camera (Manta, Allied Vision Technologies GmbH, Stadtrova, Germany) was used with a resolution of 1388 × 1032 pixels and a framerate of 10 fps. An extra illumination source with a 940–945 nm spectral range was employed to facilitate nighttime measurements. All videos were captured in raw format without any compression.

The camera was mounted on a tripod, and the system was placed in the corner of the room, strategically positioned to cover the most expansive area of where the sheep were. However, in the first two days of the experiment, a few sheep rested in Area2. It was observed that Area1 was the preferred place for them to sleep at night, so the camera was redirected to this area. As a result, during the last three days of recordings, there was a higher frequency of sheep resting in Area1.

From the videos recorded over the five days, the sheep’s rest periods were identified through a manual selection process. This process took place in two stages: in the first, there was an initial selection of the times when the sheep were resting in the designated areas, while the second stage involved a more precise selection. During rest intervals, the sheep often made movements associated with adjustments to their resting position. In the second phase, the videos were subdivided into three-minute intervals, and any segments showing movements were excluded.

The total number of hours was calculated by adding the time spent on each sheep, as the RR analysis was carried out individually for each animal. There were 35 h, 56 min, and 27 s of video with sheep in the scenes. The second selection stage, which only included moments when the sheep were at rest, lasted 29 h, 38 min, and 23 s available for analysis, representing 82.47% of coverage for RR estimation.

### 2.2. ROI Segmentation Process

#### 2.2.1. Dataset for the Training Phase

**Figure 4:**
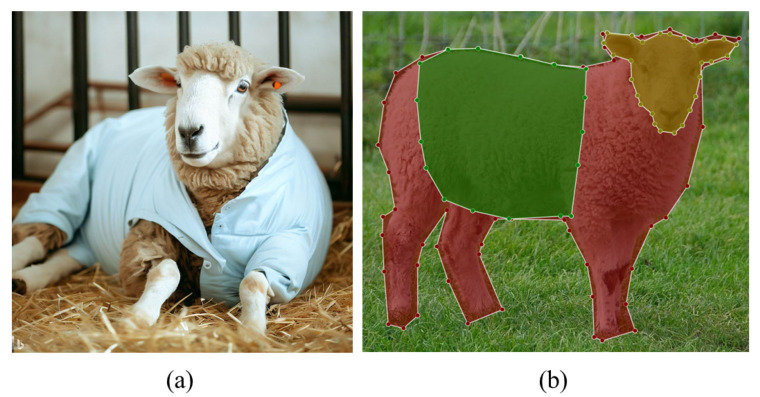
Images used during the training process. (**a**) Sheep generated artificially using the method developed by DALL-E. (**b**) Visualisation of the LabelMe tool with an annotated sheep image, where the colour representations correspond to the red area denoting the sheep, the green area representing the chest, and the yellow area indicating the face.

The images used in the training and validation phases were obtained by accessing the Flickr API [30] (591 images) and synthetically generating images using Bing Image Creator [34], powered by DALL-E [31] (200 images). These images featured sheep in a variety of contextual settings. Although many examples of sheep images were available on the Flickr API, the decision to create synthetic images was driven by the fact that the sheep in the test environment were indoors and had hospital accessories on their bodies. Figure 4a shows the result of generating synthetic images with sheep.

The annotation process was completed using LabelMe [35], a project developed by the MIT Computer Science and Artificial Intelligence Laboratory (Cambridge, MA, USA). LabelMe provides an annotation tool to build image databases to support computer vision research. The 791 images recovered were annotated manually by two experts. Figure 4b shows an example of image annotation, with manually drawn polygons corresponding to regions of interest in the sheep image, including the entire body, chest, and face.

#### 2.2.2. Segmentation Model

To segment sheep in the images and extract their corresponding thoracic regions for RR estimation, this work used the Detectron2 framework proposed by Wu et al. [36]. In addition, the authors provided access to a selection of pre-trained models trained on different datasets. In this research, we selected the Mask-R-CNN-R50-FPN architecture from these models, which had been trained on the CoCo dataset [37]. The Mask-R-CNN-R50-FPN is a deep learning model explicitly designed for instance segmentation. Its architecture includes a ResNet-50 backbone of 50 convolutional layers that extract features from the input image. These extracted features are then used within a feature pyramid network (FPN) to construct a multi-scale feature pyramid, thereby increasing the accuracy of object detection and segmentation. Throughout the training process, we followed the methodology proposed by [27], making the necessary adjustments to the specific parameters of the model. Data augmentation techniques were also used to enrich the dataset and improve the model’s performance.

The images were randomly divided into two subsets, the training and validation sets, with 80% (633 images) of the total dataset used for training and the remaining 20% (158 images) used for validation. 100,000 iterations were required to generate the final model. During training, weights were periodically adjusted to optimise learning on the validation set. The training procedure was performed using a GeForce RTX3090 Super GPU (NVIDIA Corporation, Santa Clara, CA, USA). Due to the significant amount of frames captured during the experiments (Section 2.1), the neural network evaluation stage was only applied to a representative number of frames from each collected video (158 images), maintaining the same proportion as the validation set. The frames were chosen randomly. The test set was also manually annotated using the LabelMe tool.

Applying the segmentation to the video frames results in three outputs: the certainty score ranging from 0 to 1, the binary mask, and the predicted class label. For this study, a certainty score above 0.75 was considered a valid segmentation. The intersection over union (IoU) was used as a metric to evaluate the neural network in the test set. It is based on the Jaccard Index [38], which evaluates the overlap between two bounding boxes of the real object and the predicted object. The value calculated is the percentage of the area of the overlap to the area of the union of the two objects: $IoU=Area_{intersection}/Area_{union}$. The pixel coordinates of the bounding box and the mask were provided as output to the neural network for this evaluation.

### 2.3. Respiratory Rate Estimation

One method of monitoring RR is to assess the motion generated by the chest movement during the respiratory cycle, which can be visualised by analysing the expansion and contraction of the thorax, recorded using markers that are tracked during a period [39] in the *x* and *y* planes in frames of a video. The method of extracting RR from video recordings of resting animals can be divided into five main steps, which are described in the following sections.

#### 2.3.1. Preprocessing

When performing the prediction on the first video frame, a binary mask of the found thoracic region of the sheep is provided as the network output. A multiplication function was then applied to the obtained mask and the original image, ensuring that only the thoracic region was analysed without considering other artefacts in the scene. Both the frames from the videos recorded by the sheep in the first and second stages were also enhanced using adaptive histogram equalisation. The enhancement applies the balance of grey levels in blocks within the image to enhance corners, edges and textures and to improve brightness exposure. Notably, in the second stage, in some scenes, two or more sheep were present simultaneously. This particular situation allowed the network to segment additional thoracic regions. For each identified instance, the enhancement process was performed independently.

#### 2.3.2. Motion Tracking

With the ROI determined, the algorithm proposed by Rosten et al. [40] was used to define the feature points (FPs) to be tracked. Evaluating the intensities of the neighbouring pixels as grayscale values, a pixel is an FP if its intensity is brighter or darker than its neighbours. The threshold for contrast used was 2, and the grid size was 4 × 4 pixels. The advantage of this algorithm is that it establishes the intensity check rule in only a few elements of the neighbourhood, making the detection faster. N=200 distinct FPs were selected in the ROI segmented and enhanced in the previous step. This process was used for the first frame of the video analysis and served as a parameter for the Lucas-Kanade algorithm [41], which computed the optical flow to track their trajectories over time, where it is possible to measure the apparent movement of pixels in different frames. The method returns a vector with the coordinates related to the magnitude of the pixel movement over time. These motion trajectories were subsequently separated into vertical and horizontal components. Due to the influence of rotation on the calculation of pixel trajectories in the *x* and *y* directions, the discrete derivative was applied to obtain the curve describing the magnitude of chest volume expansion over time.

#### 2.3.3. Filtering

To ensure that only respiration-related trajectories were considered, a constraint was applied to the signal frequencies to filter out noise. Sheep RR typically falls within 20–30 breaths per minute under normal conditions [14]. Therefore, a second-order Butterworth band-pass filter with a lower and upper cut-off frequency of 0.33 Hz (20 breaths/min) to 0.67 Hz (30 breaths/min) was employed to isolate the signal within the range. The sampling rates (SR) for the two experiments conducted in this study differ. In this regard, the parameters were adjusted as follows: for the first stage, SR was set at 20 fps, whereas for the second stage, SR was reduced to 10 fps. This filtering process helps to enhance the accuracy of respiration rate estimation by removing unwanted noise and focusing on the relevant respiratory components.

#### 2.3.4. Component Selection

The primary signal of interest is the chest motion induced by respiration. However, there may be additional artefacts of movement that can impact the trajectories of the FPs or even points that only have stationary trajectories, which are caused by the noise present in the image or a false FP detected in the motion tracking stage. To address this, blind source separation was implemented using principal component analysis (PCA) [42] to isolate the principal dimensions that represent the variations in chest position. Essentially, this methodology decomposes the initial dataset into a new set of variables that exhibit linearly uncorrelated relationships. For a given set of *N* FP trajectories, PCA identifies the principal components that capture the significant sources of variation. Afterwards, the fast Fourier transform (FFT) was applied to the first six components obtained from the PCA to identify which component the desired respiratory frequency was found. Due to the precise representation of the sign of contraction and relaxation of the animal’s chest, which was already evident in the first component, it was decided to consider this pattern.

#### 2.3.5. Respiratory Curve

**Figure 5:**
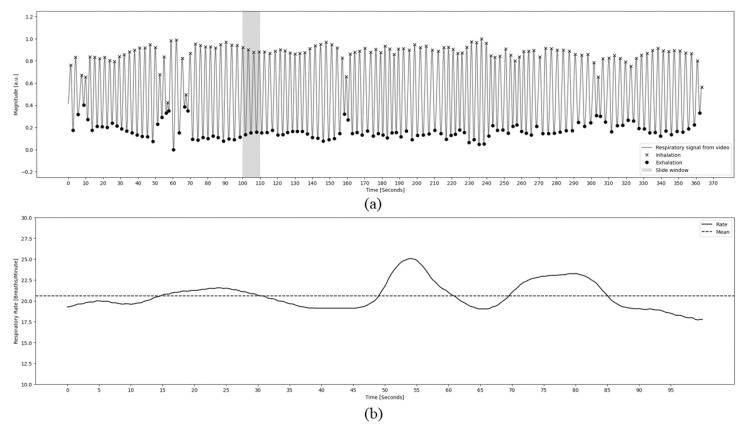
(**a**) Signal obtained through the processing of methods for acquiring the respiratory cycle in an RGB video. The graph shows the periods of inspiration and expiration caused by the thoracic movement tracked by the FP across the frames, where peaks correspond to inhalation and valleys to the exhalation of the analysed sheep. There is also a representation of the sliding window to illustrate the calculation interval of RR. (**b**) RR obtained through the processing of the respiratory cycle signal. The graph displays the rate curve and an average line over time in seconds.

The signal selected in the previous step was submitted to the RR computation process over time. An overlapping window was applied in the clipped signal in ranges of 10 s (200 frames in the first stage and 100 frames in the second stage), advancing frame by frame. Figure 5a shows the respiratory signal obtained by the computation. The detrend, zero padding, FFT, and peak detection methods were used to process and extract the dominant frequency within the specific time interval that determined the amount of inhalation and exhalation performed by the sheep. Finally, the convolution method was also applied to the resulting signal to smooth out the RR curve, obtaining the average curve. Figure 5b shows the RR resulting from processing.

Figure 5a shows clearly the cyclical/sinusoidal movements of the chest during the respiration cycle, where the upper peaks represent the maximum expansion of the abdomen after inspiration and the valleys the maximum relation of the muscles after expiration. In this sense, it was possible to estimate the respiratory rate, as shown in Figure 5b.

#### 2.3.6. Evaluation—First Stage of Study

RR estimation was performed in these regions to investigate the influence of respiratory signal strength in the different areas of the sheep thorax. From the segmented image obtained in Section 2.3.1, three sections were defined, with R1 corresponding to the deepest abdominal region, R2 to the middle area, and R3 to the thoracic region closest to the animal’s head, as illustrated in the Figure 2. To assess the agreement between the results computed by the algorithm and the real RR value, four experts were requested to visually count the respiratory movements of the sheep for the given video sequences.

#### 2.3.7. Evaluation—Second Stage of Study

Due to the amount of recording hours obtained in the second experiment, only one expert participated in counting the chest movements of these animals. Since the sheep were in their usual environment, and no human interference occurred during the capture days, it was assumed that the RR would remain relatively unchanged in 3-min intervals. Therefore, the counting was only conducted during the first minute of each block.

## 3. Results

### 3.1. Segmentation

**Figure 6:**
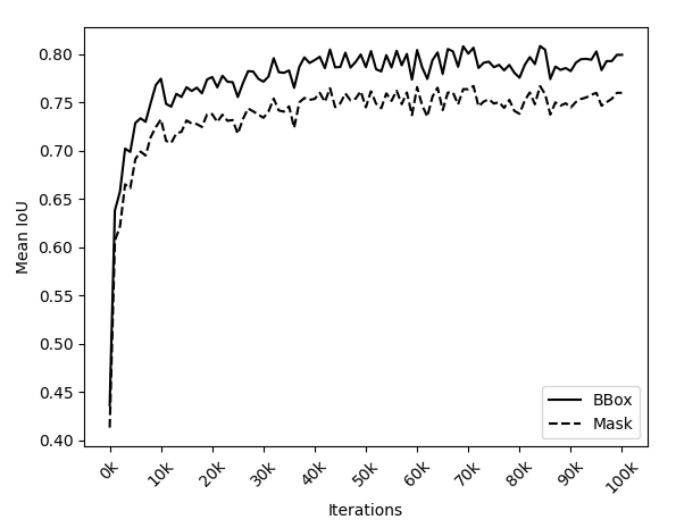
IoU in the validation set during the iterations of the Detectron2 training process.

Figure 6 shows the learning curve obtained from the training process during the model segmentation generation phase. The graph displays two curves: the solid line represents the mean IoU metric for the bounding box over the iterations on the validation set, while the dotted line indicates the mean IoU for the mask. It can be observed from this representation that the learning rate reaches an average IoU Box of approximately 70% within the first 10,000 iterations, fluctuating up to around 80% until the end of iteration processing. The IoU averages were calculated as the mean across the three detected (IoU BBox) and segmented (IoU Mask) classes: whole body, face, and thorax of the sheep in the validation set (from Flick API and DALL.E).

Similar behaviour can be observed between the curves due to Detectron2 providing results in two outputs: one for the detected region and another for the segmented object. The distance between these curves indicates some discrepancies observed in the mask, as the segmentation is more sensitive to changes in the image, considering the object’s shape rather than just the region it occupies.

To ensure effective detection in the test set, an IoU Box threshold of 80% was applied; this means that objects detected by the network with an IoU value above 80% were classified as valid ROIs. Table 1 presents the results obtained after the segmentation step for RGB and NIR images. The Object column lists each instance evaluated during segmentation. IoU BBox indicates the IoU percentage for the bounding box, while IoU Mask represents the IoU percentage for the mask. The certainty score is the confidence metric the neural network provides. The IoU for bounding boxes (IoU BBox) showed an average coverage of 84.95%, whereas the IoU for masks (IoU Mask) averaged 69.37%.

**Figure 7:**
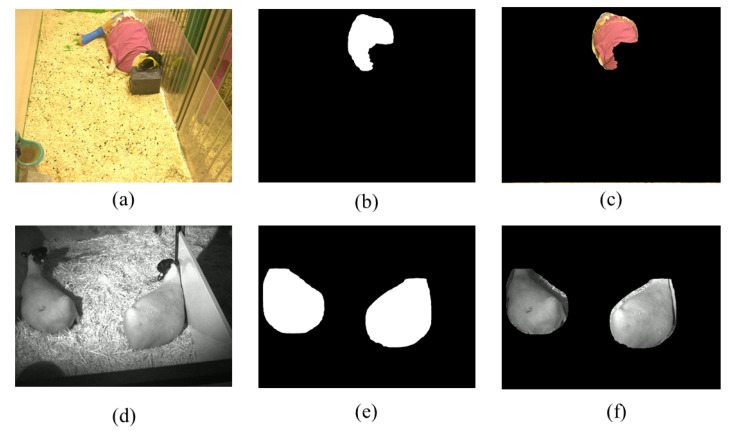
Process of delimiting the sheep’s thoracic region to acquire the RR using the generated Detectron2 model. (**a**) RGB original image. (**b**) RGB binary mask. (**c**) RGB segmented thoracic region. (**d**) NIR original image. (**e**) NIR binary mask. (**f**) NIR segmented thoracic region.

Figure 7 illustrates the segmentation results for the thoracic region in both the RGB and NIR video stages. The six subfigures represent the ROI extraction process, where RR extraction methods were applied. Figure 7a shows a frame captured by the RGB camera featuring a single sheep, Figure 7b presents the binary mask generated by Detectron2, and Figure 7c illustrates the fully segmented region. Notably, the ROI was automatically segmented, accounting for artefacts such as dressings and coverings on the animal’s body. This outcome is due to the Detectron2 training, which considered post-surgical scenarios commonly encountered in laboratory experiments involving ovine models.

The same process was applied to the NIR images: Figure 7d shows a frame from the NIR video set with two sheep, Figure 7e displays the binary segmentation mask, and, finally, Figure 7f highlights the thoracic regions of both sheep segmented simultaneously. This section highlights the network’s performance in segmenting the thoracic region, as the study focuses on analysing respiratory movement detected through motion in this area. Although effective, the segmentation of the face and body will serve as a basis for future studies and vital signal measurement.

### 3.2. Estimation of Respiration Rate

#### 3.2.1. First Stage—RGB Videos

Table 2 presents the results obtained for the first stage of the study (Section 2.1.1), where RGB videos were acquired from one sheep. The ID_V_ identifies the specific video on which the evaluation was conducted. Duration refers to the length of each video, measured in minutes and seconds. RR_Ref_ represents the mean RR values acquired by the specialists, expressed in breaths per minute. The ROI column indicates the RR calculation derived from the chest region (Total) and sub-regions (R1, R2, and R3 represented in Figure 2). RR_Video_ represents the RR values obtained through the proposed method, measured in breaths per minute. Finally, the columns $\bar{\varepsilon }$ and ${\bar{\varepsilon }}_{r}$ present both the absolute and relative errors when comparing the RR values obtained from the specialists (RR_Ref_) and the RR_Video_, respectively.

The mean obtained for RR_Ref_ in the total ROI was 28.88 ± 4.41, and for RR_Video_ 28.62 ± 3.76, both in breaths/min. The mean absolute error observed was 0.79 in breaths/min. Distinguishing the regions, the mean absolute errors were 1.01 breaths/min for R1, 1.09 breaths/min for R2, and 2.32 breaths/min for R3.

**Figure 8:**
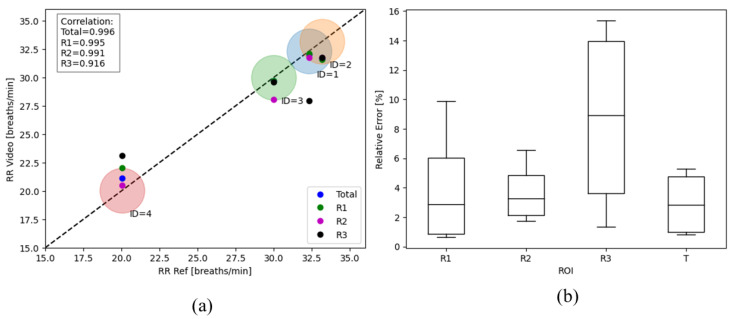
(**a**) Correlation plot comparing RR_Ref_ and RR_Video_ distinguishing the three ROIs. The ID specified in the plot refers to the processed video. (**b**) Box plot showing the relative errors obtained by each ROI and the total area.

The agreement between the two metrics was calculated with the correlation considering three scenarios: total chest, R1, R2, and R3. Figure 8a shows the graph of the RR results obtained versus the ground truth (GT) of RR. The RR_Video_ considering the total area showed the highest agreement, measuring 0.996. Looking at the metric correlation between the ROIs, it is noticeable that R3 (0.916) has a lower correlation than the others. In addition, Figure 8b presents the box plot of the relative errors obtained for each individually measured region in the signal intensity analysis. The smallest errors were obtained when the analysis was performed over the entire chest; however, when comparing the three areas, R1 and R2 showed lower means and deviations than R3.

The errors observed in R3 suggest that the expansion resulting from RR exerts a greater influence on the abdominal than the thoracic region of the animal considering the movement. This analysis indicates that the entire ROI, particularly the R1 and R2 regions, are more likely to provide a clearer RR signal, as they show more significant correlations with the movement of the respiratory cycle. It is also noteworthy that the sheep exhibited sudden movements during the extraction of the RR data, which were also tracked by the optical flow. This was reflected in the resulting RR signal, resulting in some inconsistencies and an increased percentage of relative errors.

#### 3.2.2. Second Stage—NIR Videos

This section presents the results by analysing the NIR videos obtained in the second stage of the study (Section 2.1.2). The main aim was to check the algorithm’s feasibility when multiple animals are present in the same image and to study its robustness to different long-term measurements, conditions, and body positions. Table 3 presents the observation period, which lasted 29 h, 38 min, and 23 s. The column labelled as Area is denoted as the location where the camera was positioned. The Day column indicates the day the video was captured. The ID_V_ identifies the specific video on which the evaluation was conducted. Duration refers to the length of each video, measured in hours, minutes, and seconds. The ID_S_ was designed to count the sheep in the video. It is important to emphasise that no animal identification procedures were carried out. Rather, the ID_S_ is intended to highlight the number of animals in the video sequence. It is also worth noting that the camera was randomly placed in either Area1 or Area2, as the spatial conditions did not allow data to be collected from both areas simultaneously. RR_Ref_ represents the mean RR values acquired by the specialist, expressed in breaths per minute. RR_Video_ represents the RR values obtained through the proposed method, measured in breaths per minute. $\bar{\varepsilon }$ is the mean absolute error, and ${\bar{\varepsilon }}_{r}$ is the mean relative error. In this phase of the study, the entire ROI of the sheep models was analysed without considering specific regions as was the case in the first study (Section 2.1.1).

The results demonstrated that the errors obtained when comparing RR_Ref_ and RR_Video_ in NIR experiments, where the mean of RR_Ref_ was 28.85 ± 3.34 and RR_Video_ was 29.79 ± 2.40, in breaths/min. The mean relative error was 6.39 ± 3.70.

**Figure 9:**
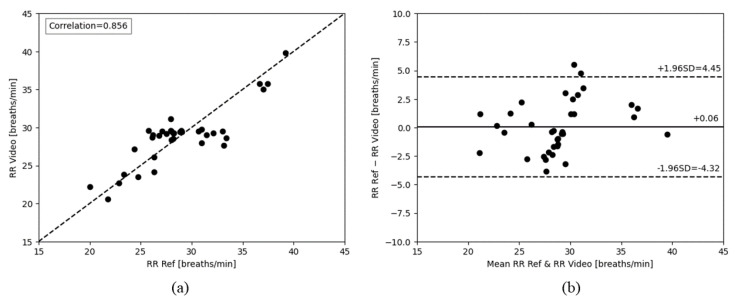
(**a**) Correlation plot comparing RR_Ref_ and RR_Video_ acquired by processing NIR videos. (**b**) Bland-Altman plot comparing RR_Ref_ and RR_Video_ acquired by processing NIR videos.

The analysis also revealed an RR correlation of 0.856, as shown in Figure 9a. The Bland-Altman [43] plot in Figure 9b showed a mean difference of 0.06 breaths/min. The limits of agreement ranged from −4.32 to 4.45 breaths/min.

During the acquisition of the NIR videos, some queries were raised regarding the location of the sheep during the collection period. It was observed that the majority of the time, the sheep did not remain in Area2 but instead selected Area1 as a resting location, as illustrated in Table 3. Consequently, the decision was taken to reposition the camera on specific days, resulting in a considerably greater quantity of video footage being available for analysis on days D3, D4, and D5. This emphasises the necessity of strategically selecting the camera position to ensure comprehensive and representative data collection. During the selection of videos for analysis, a disparity was observed in the availability of valid data for applying the RR extraction algorithm. Focusing only on the periods when the sheep were resting, an interesting trend was observed in the timeline of the five days of acquisition: the sheep tended to remain in this state for longer at night. This was to be expected, given that the sheep were in their natural environment (barn), rather than in a laboratory setting.

## 4. Discussion

This research paper aims to demonstrate the capability of extracting the RR of sheep by analysing their chest movements, which are recorded on video. Measuring the RR is essential for the assessment of health status and physiological deterioration not only for humans but also for laboratory animals [28]. Several studies have shown the advantages of using sheep as an animal model, as they are similar to human organs. However, submitting sheep to experimental conditions can cause discomfort, pain, and stress. Therefore, suggesting methods that capture vital signs, such as RR, in a non-invasive and utterly non-contact manner becomes essential when aiming to adhere to the principles of the 3Rs. The relationship between the computational technologies available today and the problems inherent in biomedical laboratory practice has also been the focus of research groups working to improve animal welfare. In the literature, as mentioned before, some studies highlight the relationship between RR variations and pain, stress, and discomfort levels in sheep. The main objective of the methodologies employed is to improve techniques and ensure a continuous animal welfare assessment. Monitoring vital signs, such as RR and heart rate, provides insight into the animal’s condition, allowing the responsible professionals to understand the situation and take the necessary action. The technologies available today make it possible to innovate and apply these techniques to evaluate signals of this nature.

In this context, the study proposed a methodology based on automated digital image processing that included the following steps: new data acquisition (RGB and NIR images), segmentation of the ROI using deep learning techniques, detection of FP, motion tracking of pixels over time to obtain optical flow, and finally processing of the signal to obtain the RR. As a proof of concept, the proposed algorithm was applied in two scenarios, the first with one sheep and RGB videos and the second with five sheep and NIR videos.

In the segmentation phase, some studies proposed models that detect and segment animals since animal images are widely available to the scientific community. The Flickr [30] provides an API that can retrieve images containing specific objects by querying its image database. In addition to the images, ref. [37] also provides polygon annotations that delineate the region in the image where particular objects are located. On the other hand, this tool did not provide the ROI required for this study, which led to the use of LabelMe software [35] on the images obtained through the [30]. These ROIs were the entire body of the sheep, the thoracic region, and the face.

Some conditions made this first analysis challenging during the segmentation stage, as the sheep was wearing surgical clothing, and the available image datasets with sheep only had examples of sheep without props and in a non-laboratory environment. This meant that the neural network did not have enough descriptive input images to train the segmentation model for the region corresponding to the sheep’s chest. This led to the search for image generation via DALL.E, where it was possible to generate images of sheep with artefacts and effectively perform neural network training and segmentation. In this context, generating artificial images has proven to be essential to generating models in specific scenarios for automated segmentation.

To segment the sheep thorax in video frames from the RGB camera, it was necessary to use 200 artificially generated images to create a model capable of segmenting the sheep’s thoracic region. Both stages, RGB and NIR images, were included in the test set. IoU Bbox (84.45 ± 1.30) obtained during the evaluation on the test set proved to be satisfactory for our application scenario since an IoU of more than 50% is generally considered a valid detection. However, it is possible to notice a decrease when looking at the IoU obtained for the mask (69.37 ± 1.14). This is due to the refinement provided by the polygon segmentation of the image, where small discrepancies between the manual annotation and the segmentation lead to a reduction in the values.

Qin and co-authors [44] proposed a method to measure the body structures of Ujumqin sheep using images to assess their growth and development. Images were taken of the sheep in a controlled environment, capturing them from both angles, from above and from the side, while it was standing. This work also used the Mask R-CNN architecture. An image calibration step was included to establish the scale relationship between pixels and distance. The study captured the region of the sheep’s chest, but it did not present a method to obtain exclusively the ROI of the chest since the measurements obtained were based on the entire mask of the sheep in a given position.

In the field of image-based vital signs detection, different publications [22,26,27,28,29] mention the use of video frame processing techniques and motion tracking analysis to extract RR, demonstrating the accuracy of methods (Lukas-Kanade, FFT, and PCA) for estimating vital signs such as RR and heart rate (HR), showing results comparable to those from established devices like photoplethysmography (PPG) and electrocardiography (ECG). This technique has been successfully tested for measuring these signs in humans and small and large animals. It is worth noting that the proposed study aims to assess the condition of sheep under experimental settings to contribute to the development of contactless monitoring systems for these animal models. This approach is intended to enhance animal welfare by minimising (aligning with 3Rs) the need for invasive contact that some devices may require.

Only the study conducted by Fuentes et al. [22] discusses obtaining vital signals in sheep by analysing video footage. The authors developed a method capable of extracting biometric signals (RR, HR, and temperature) from sheep in a transport environment. The face and nostril regions were defined as the ROI for signal measurement. To calculate the respiratory and heart rate metrics, the authors utilised variations in brightness within the ROI in RGB and infrared video frames. They obtained a correlation of 0.94 when considering both signals. Additionally, they employed machine learning techniques to classify the RR of the sheep into low, medium, or high categories. However, this study has some notable limitations. Primarily, it is restricted to measurements in fully controlled environments (transportation ovine setup). In addition, it is based on signal acquisition from the nasal region, and if the sheep’s face is not visible, the proposed system can not measure its vital signs. Such elements can be subject to interference due to the environmental conditions in which the analysis is being conducted and the thermal and metabolic variations among individual animals. Another factor to consider is the sheep’s fleece density. Depending on the thickness of the wool layer, the temperature measurement in certain areas might be inaccessible or unreliable.

The measurement of the RR through the analysis of sheep’s chest movement (our proposal) offers distinct advantages within the context of vital signs monitoring. This is due to the reduced need for additional hardware, as many devices already incorporate cameras to capture images. This eliminates the need for additional sensors and avoids further physical contact with the animal. In addition, this approach is advantageous from a cost-benefit standpoint because it does not require specialised hardware to acquire these signals, making it an economical solution compared to traditional veterinary equipment.

## 5. Conclusions

This study has demonstrated a methodology capable of detecting and segmenting RGB and NIR scenarios. This study provides an image base with polygonal annotations of the whole body, face, and chest regions that can be used to train new models and contribute to further research. The errors showed a significant agreement with the measurements obtained by the experts. It is also important to observe that 82.47% of the sheep videos were successfully processed in the second stage, demonstrating the high capacity of the method to evaluate signals in less controlled scenarios, unlike the studies mentioned in the literature.

The limitations of this study concerning the segmentation stage are related to the inability of the model to deal with situations where only part of the sheep is visible in the image, rather than the whole body. In some cases, the performance of the model was found to be unsatisfactory in these scenarios. Another limitation of this study, as identified in the paper, is the absence of a GT given by a sensor, such as PPG. Regrettably, any experiment involving direct contact with animals, such as the use of a sensor, necessitates approval from the relevant authority. This process can entail several months of paperwork. However, this requirement does not extend to imaging techniques, as they are non-contact methods and do not cause any disruption. Despite the lack of GT, Figure 5a demonstrates that the signal extracted from the camera aligns with the anticipated respiratory waveform. Furthermore, the respiratory expansion is so pronounced in the video that each breath can be counted merely by observing the video sequences. Consequently, we employed manual counting as a reference, considering it an appropriate method for assessing this parameter.

For future research, the method can be improved by incorporating features that can detect the behaviour of the sheep (whether they are lying down or standing, as was carried out by [45,46]) and, based on this, measure the RR. This approach would eliminate the need for the manual motion analysis step performed in this study. An additional perspective for further development would be to analyse RR at different video compression levels. This is justified by the fact that, although the resolution used in this study is of high quality, generalising the solution to different cameras and levels of image quality would be ideal. This approach would allow the solution to be used effectively by veterinary and farm professionals, regardless of variations in image quality. In the context of RR analysis, this solution enables professionals to make early decisions when faced with cases of infection and disease in animals. The study presented also provides methods that can be applied to different types of animals, requiring only the adaptation of the MaskRCNN segmentation model. This makes the method viable for use with cattle, horses, pigs and other mammalian species.

## Figures and Tables

**Table 1:** Sheep Detection and Segmentation Results.

Object	IoU BBox	IoU Mask	Certainty Score
Sheep	86.90 ± 2.84	67.73 ± 16.90	95.91 ± 1.89
Face	84.55 ± 3.15	71.08 ± 14.54	98.89 ± 1.19
Thorax	83.40 ± 3.38	69.30 ± 17.33	96.71 ± 2.09
Mean ± SD	84.95 ± 1.30	69.37 ± 1.14	97.17 ± 1.15

IoU Box, Mask and Certainty Score - %.

**Table 2:** Estimation of RR using video recordings from the RGB camera.

ID_Video_	Duration	$\bar{\mathrm{RR}}$ _Ref_	ROI											
			**Total**			**R1**			**R2**			**R3**		
			$\bar{\mathrm{RR}}$ _**Video**_	$\bar{\varepsilon }$	${\bar{\varepsilon }}_{r}$	$\bar{\mathrm{RR}}$ _**Video**_	$\bar{\varepsilon }$	${\bar{\varepsilon }}_{r}$	$\bar{\mathrm{RR}}$ _**Video**_	$\bar{\varepsilon }$	${\bar{\varepsilon }}_{r}$	$\bar{\mathrm{RR}}$ _**Video**_	$\bar{\varepsilon }$	${\bar{\varepsilon }}_{r}$
1	00:26	32.31	32.05	0.26	0.80	32.10	0.21	0.64	31.75	0.56	1.73	27.96	4.35	13.46
2	00:26	33.17	31.65	1.52	4.59	31.59	1.58	4.77	31.76	1.41	4.26	31.72	1.45	4.38
3	00:26	30.00	29.69	0.31	1.03	29.72	0.28	0.93	28.04	1.96	6.53	29.60	0.40	1.33
4	06:03	20.04	21.10	1.06	5.28	22.02	1.98	9.87	20.49	0.45	2.24	23.12	3.08	15.36
Mean		28.88	28.62	**0.79**	2.93	28.86	**1.01**	4.06	28.01	**1.09**	3.69	28.10	**2.32**	8.63
±SD	-	4.42	3.76	0.50	2.01	3.42	0.77	3.27	3.76	0.59	1.71	2.56	1.39	5.78

$\bar{RR}$ and $\bar{\varepsilon }$—[breaths/min], ${\bar{\varepsilon }}_{r}$—%.

**Table 3:** Estimation of RR using video recordings from the NIR camera.

Area	Day	ID_Video_	ID_Sheep_	Duration	$\bar{\mathrm{RR}}$ _Ref_	$\bar{\mathrm{RR}}$ _Video_	$\bar{\varepsilon }$	${\bar{\varepsilon }}_{r}$
Area1	Day 1	1	1	00:58:47	31.47	29.00	2.47	7.85
		2	1	00:37:52	26.15	28.70	2.55	9.75
	Day 2	3	1	00:45:00	23.33	23.79	0.46	1.97
		4	1	00:08:00	26.33	24.11	2.22	8.43
Area2	Day 3	5	1	00:48:00	25.72	29.75	3.82	14.83
		6	1	01:21:00	28.22	29.23	1.01	3.58
			2	01:21:00	24.37	27.13	2.76	11.33
		7	1	00:12:00	31.00	27.99	3.01	9.71
	Day 4	8	1	01:03:00	27.52	29.20	1.68	6.10
			2	01:09:00	28.04	29.49	1.45	5.17
		9	1	02:04:00	28.88	29.42	0.54	1.87
			2	01:58:20	29.03	29.40	0.37	1.27
		10	1	02:10:00	28.27	29.26	0.99	3.50
			2	00:45:00	27.93	29.55	1.62	5.80
		11	1	00:53:00	26.33	26.09	0.24	0.91
		12	1	00:08:00	30.67	29.48	1.19	3.88
		13	1	00:40:00	28.21	28.51	0.30	1.60
		14	1	00:36:00	26.17	28.99	2.82	10.78
		15	1	00:45:00	31.13	27.93	3.20	10.28
			2	01:22:00	37.43	35.73	1.70	4.54
			3	00:51:00	22.88	22.71	0.17	0.74
	Day 5	16	1	00:16:00	39.17	39.78	0.61	1.56
			2	00:10:00	21.75	20.55	1.20	5.52
		17	1	00:57:00	30.95	29.78	1.17	3.78
			2	00:54:00	26.78	28.94	2.16	8.07
		18	1	00:36:00	27.08	29.48	2.40	8.86
			2	00:21:00	28.00	28.40	0.40	1.43
		19	1	00:40:00	33.39	28.63	4.76	14.26
		20	1	01:32:16	36.67	35.76	0.91	2.48
		21	1	00:03:00	37.00	34.99	2.01	5.43
		22	1	00:20:00	33.00	29.52	3.48	10.55
		23	1	00:40:00	33.14	27.62	5.52	16.66
			2	00:36:00	29.00	29.57	0.57	1.97
		24	1	00:12:00	24.75	23.51	1.24	5.01
			2	00:12:00	20.00	22.20	2.20	11.00
		25	1	01:33:00	32.13	29.28	2.85	8.87
Mean					28.85	28.79	1.83	6.39
±SD					3.34	2.40	1.05	3.70

$\bar{RR}$ and $\bar{\varepsilon }$—[breaths/min], ${\bar{\varepsilon }}_{r}$—%.

## Data Availability

The data presented in this study is available on request from the corresponding author. The data are not publicly available due to the file size of the raw videos.

## Abbreviations

The following abbreviations are used in this manuscript:

Area1	Area of video acquisition 1
Area2	Area of video acquisition 2
BBox	Bounding Box
ECG	Electrocardiography
FFT	Fast Fourier Transform
FPN	Feature Pyramid Network
FPs	Feature Points
fps	frames per second
GT	Ground Truth
HR	Heart Rate
Hz	Hertz
ID_Sheep_	Sheep Model Unique Identifier
ID_Video_	Video Unique Identifier
IoU	Intersection over Union
m	meters
min	minutes
N	Number of samples
NIR	Near-Infrared
nm	nanometer
PCA	Principal Component Analysis
PPG	Photoplethysmography
R1	Region of Interest of the Sheep Thorax in Position 1
R2	Region of Interest of the Sheep Thorax in Position 2
R3	Region of Interest of the Sheep Thorax in Position 3
RGB	Red, Green and Blue
ROI	Region of Interest
RR	Respiration Rate
RR_Ref_	Respiratory Rate from Truth Reference
RR_Video_	Respiratory Rate from Video Processing
SD	Standard Deviation
SR	Sampling Rate
T	Region of Interest of the Sheep Thorax in Total Area

## References

[1] Murray S.J., Mitchell N.L. (2022). The translational benefits of sheep as large animal models of human neurological disorders. Front. Vet. Sci..

[2] Sartoretto S.C., Uzeda M.J., Miguel F.B., Nascimento J.R., Ascoli F., Calasans-Maia M.D. (2016). Sheep as an experimental model for biomaterial implant evaluation. Acta Ortop. Bras..

[3] Turner A.S. (2007). Experiences with sheep as an animal model for shoulder surgery: Strengths and shortcomings. J. Shoulder Elb. Surg..

[4] Franco N.H. (2013). Animal experiments in biomedical research: A historical perspective. Animals.

[5] Broom D.M. (2011). A history of animal welfare science. Acta Biotheor..

[6] Prescott M.J., Lidster K. (2017). Improving quality of science through better animal welfare: The NC3Rs strategy. Lab Anim..

[7] Parlamient E. (2010). Directive 2010/63/EU of the European Parliament and of the Council of 22 September 2010 on the protection of animals used for scientific purposes. Off. J. Eur. Union.

[8] Russell W.M.S., Burch R.L. (1959). The Principles of Humane Experimental Technique.

[9] Commission E. (2020). 2019 Report on the Statistics on the Use of Animals for Scientific Purposes in the Member States of the European Union in 2015–2017. https://op.europa.eu/en/publication-detail/-/publication/04a890d4-47ff-11ea-b81b-01aa75ed71a1.

[10] Banstola A., Reynolds J.N. (2022). The sheep as a large animal model for the investigation and treatment of human disorders. Biology.

[11] Nikita K.S. (2014). Handbook of Biomedical Telemetry.

[12] Mösch L., Kunczik J., Breuer L., Merhof D., Gass P., Potschka H., Zechner D., Vollmar B., Tolba R., Häger C. (2023). Towards substitution of invasive telemetry: An integrated home cage concept for unobtrusive monitoring of objective physiological parameters in rodents. PLoS ONE.

[13] Jackson P.G.G., Cockcroft P.D. (2002). Clinical Examination of the Respiratory System.

[14] Reece W.O. (2015). Overview of the respiratory system. Dukes’ Physiology of Domestic Animals.

[15] Nagy D.W., Pugh D. (2012). Handling and examining sheep and goats. Sheep and Goat Medicine.

[16] Reefmann N., Kaszàs F.B., Wechsler B., Gygax L. (2009). Physiological expression of emotional reactions in sheep. Physiol. Behav..

[17] De K., Saxena V.K., Balaganur K., Kumar D., Naqvi S.M.K. (2018). Effect of short-term seclusion of sheep on their welfare indicators. J. Vet. Behav..

[18] Cwynar P., Kolacz R. (2011). The effect of sound emmission on sheep welfare. Proceedings of the Animal Hygiene and Sustainable Livestock Production. XVth International Congress of the International Society for Animal Hygiene.

[19] Knight M.I., Linden N.P., Butler K.L., Rice M., Ponnampalam E.N., Behrendt R., Jongman E.C. (2023). The effect of shade on sheep grazing pasture during summer conditions. J. Vet. Behav..

[20] Cui Y., Zhang M., Li J., Luo H., Zhang X., Fu Z. (2019). WSMS: Wearable stress monitoring system based on IoT multi-sensor platform for living sheep transportation. Electronics.

[21] Strutzke S., Fiske D., Hoffmann G., Ammon C., Heuwieser W., Amon T. (2019). Development of a noninvasive respiration rate sensor for cattle. J. Dairy Sci..

[22] Fuentes S., Gonzalez Viejo C., Chauhan S.S., Joy A., Tongson E., Dunshea F.R. (2020). Non-invasive sheep biometrics obtained by computer vision algorithms and machine learning modeling using integrated visible/infrared thermal cameras. Sensors.

[23] Balakrishnan G., Durand F., Guttag J. Detecting pulse from head motions in video. Proceedings of the IEEE Conference on Computer Vision and Pattern Recognition.

[24] Monkaresi H., Calvo R.A., Yan H. (2013). A machine learning approach to improve contactless heart rate monitoring using a webcam. IEEE J. Biomed. Health Inform..

[25] Wang H., Huang J., Wang G., Lu H., Wang W. Surveillance Camera-based Cardio-respiratory Monitoring for Critical Patients in ICU. Proceedings of the 2022 IEEE-EMBS International Conference on Biomedical and Health Informatics (BHI).

[26] Barbosa Pereira C., Dohmeier H., Kunczik J., Hochhausen N., Tolba R., Czaplik M. (2019). Contactless monitoring of heart and respiratory rate in anesthetized pigs using infrared thermography. PLoS ONE.

[27] Breuer L., Mösch L., Kunczik J., Buchecker V., Potschka H., Czaplik M., Pereira C.B. (2023). Camera-Based Respiration Monitoring of Unconstrained Rodents. Animals.

[28] Barbosa Pereira C., Kunczik J., Zieglowski L., Tolba R., Abdelrahman A., Zechner D., Vollmar B., Janssen H., Thum T., Czaplik M. (2018). Remote welfare monitoring of rodents using thermal imaging. Sensors.

[29] Jorquera-Chavez M., Fuentes S., Dunshea F.R., Warner R.D., Poblete T., Jongman E.C. (2019). Modelling and validation of computer vision techniques to assess heart rate, eye temperature, ear-base temperature and respiration rate in cattle. Animals.

[30] Flickr API. https://www.flickr.com/services/api/.

[31] OpenAI DALL·E: Creating Images from Text. https://openai.com/research/dall-e.

[32] Digging into Detectron 2—Part 1. https://medium.com/@hirotoschwert/digginginto-detectron-2-47b2e794fabd.

[33] Council N.R. (2011). Guide for the Care and Use of Laboratory Animals.

[34] Microsoft Bing Image Creator. https://www.bing.com/create.

[35] Wada K. Labelme: Image Polygonal Annotation with Python. https://github.com/wkentaro/labelme.

[36] Wu Y., Kirillov A., Massa F., Lo W.Y., Girshick R. Detectron2. https://github.com/facebookresearch/detectron2.

[37] Lin T.Y., Maire M., Belongie S., Hays J., Perona P., Ramanan D., Dollár P., Zitnick C.L. (2014). Microsoft coco: Common objects in context. Proceedings of the Computer Vision–ECCV 2014: 13th European Conference.

[38] Padilla R., Netto S.L., Da Silva E.A. A survey on performance metrics for object-detection algorithms. Proceedings of the 2020 International Conference on Systems, Signals and Image Processing (IWSSIP).

[39] Massaroni C., Nicolo A., Sacchetti M., Schena E. (2020). Contactless methods for measuring respiratory rate: A review. IEEE Sensors J..

[40] Rosten E., Porter R., Drummond T. (2008). Faster and better: A machine learning approach to corner detection. IEEE Trans. Pattern Anal. Mach. Intell..

[41] Lucas B.D., Kanade T. An iterative image registration technique with an application to stereo vision. Proceedings of the IJCAI’81: 7th International Joint Conference on Artificial Intelligence.

[42] Abdi H., Williams L.J. (2010). Principal component analysis. Wiley Interdiscip. Rev. Comput. Stat..

[43] Bland J.M., Altman D. (1986). Statistical methods for assessing agreement between two methods of clinical measurement. Lancet.

[44] Qin Q., Dai D., Zhang C., Zhao C., Liu Z., Xu X., Lan M., Wang Z., Zhang Y., Su R. (2022). Identification of body size characteristic points based on the Mask R-CNN and correlation with body weight in Ujumqin sheep. Front. Vet. Sci..

[45] Xu J., Wu Q., Zhang J., Tait A. Automatic sheep behaviour analysis using mask r-cnn. Proceedings of the 2021 Digital Image Computing: Techniques and Applications (DICTA).

[46] Ren K., Karlsson J., Liuska M., Hartikainen M., Hansen I., Jørgensen G.H. (2020). A sensor-fusion-system for tracking sheep location and behaviour. Int. J. Distrib. Sens. Netw..

